# Enhancer trap lines with GFP driven by *smad6b* and *frizzled1* regulatory sequences for the study of epithelial morphogenesis in the developing zebrafish inner ear

**DOI:** 10.1111/joa.13845

**Published:** 2023-02-06

**Authors:** Davide Baldera, Sarah Baxendale, Nicholas J. van Hateren, Mar Marzo, Emily Glendenning, Fan‐Suo Geng, Kazutomo Yokoya, Robert D. Knight, Tanya T. Whitfield

**Affiliations:** ^1^ School of Biosciences University of Sheffield Sheffield UK; ^2^ Brain and Mind Research Institute, University of Sydney Sydney New South Wales Australia; ^3^ Centre for Craniofacial and Regenerative Biology, King's College London, Guy's Hospital London UK; ^4^ Present address: CeSASt, University of Cagliari Cagliari Italy; ^5^ Present address: Data Science Institute, The University of Technology Sydney Sydney Australia

**Keywords:** enhancer trap line, *frizzled1*, organogenesis, otic vesicle, *smad6b*, targeted locus amplification, zebrafish

## Abstract

Live imaging in the zebrafish embryo using tissue‐specific expression of fluorescent proteins can yield important insights into the mechanisms that drive sensory organ morphogenesis and cell differentiation. Morphogenesis of the semicircular canal ducts of the vertebrate inner ear requires a complex rearrangement of epithelial cells, including outgrowth, adhesion, fusion and perforation of epithelial projections to generate pillars of tissue that form the hubs of each canal. We report the insertion sites and expression patterns of two enhancer trap lines in the developing zebrafish embryo, each of which highlight different aspects of epithelial cell morphogenesis in the inner ear. A membrane‐linked EGFP driven by *smad6b* regulatory sequences is expressed throughout the otic epithelium, most strongly on the lateral side of the ear and in the sensory cristae. A second enhancer trap line, with cytoplasmic EGFP driven by *frizzled1* (*fzd1*) regulatory sequences, specifically marks cells of the ventral projection and pillar in the developing ear, and marginal cells in the sensory cristae, together with variable expression in the retina and epiphysis, and neurons elsewhere in the developing central nervous system. We have used a combination of methods to identify the insertion sites of these two transgenes, which were generated through random insertion, and show that Targeted Locus Amplification is a rapid and reliable method for the identification of insertion sites of randomly inserted transgenes.

## Graphical abstract

Baldera, Baxendale, et al. describe two enhancer trap lines that show the expression of GFP in the developing zebrafish inner ear. These lines are driven by regulatory elements at the *smad6b* and *fzd1* loci, and will be useful for imaging the dynamic events of epithelial morphogenesis that generate this intricate sensory organ system.

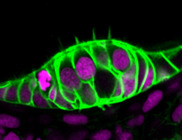

## 1 INTRODUCTION

Morphogenesis of the complex labyrinth of the vertebrate inner ear from a simple ball of epithelial cells exemplifies many core developmental processes (reviewed in [Alsina & Whitfield, 2017]). In zebrafish, the formation of the three semicircular canal ducts involves the formation of finger‐like projections of otic epithelium that grow into the lumen of the otic vesicle, where they adhere, fuse, and perforate to form pillars of tissue (Waterman & Bell, 1984). Each pillar subsequently widens to contribute to the inward‐facing wall of the curved semicircular canal duct. To understand these dynamic processes at the tissue and cellular level, it is most informative to image them in the live embryo, in real‐time. Techniques such as light‐sheet microscopy are able to image gently over long time courses with low photobleaching (Huisken et al., 2004; Power & Huisken, 2017), and the use of spinning disk confocal or Airyscan technologies allow visualisation of subcellular details in live specimens (Lam et al., 2014; Wu & Hammer, 2021).

A variety of transgenic lines have been successfully exploited for live imaging of the developing zebrafish inner ear. The ubiquitous *actb2* promoter drives transgene expression in all cells of the embryo, including the otic epithelium, and the use of tags on fluorescent proteins, or fusion to other proteins, can be used to highlight different subcellular compartments (Mosaliganti et al., 2019; Munjal et al., 2021; Swinburne et al., 2018). Promoters for *cl* *dnb* (Haas & Gilmour, 2006), *sp7* (DeLaurier et al., 2010), and *sox10* (Carney et al., 2006) can also be used to drive transgene expression throughout the developing otic vesicle. A few cell‐type‐specific transgenic lines are available for studying the developing ear in zebrafish, including those utilising promoters that drive expression in neuronal progenitors (*isl2; neurod*), sensory hair cells (*pou4f3; myo6b*) and supporting cells (*agr2*) (reviewed in [Baxendale & Whitfield, 2016]). The identification of additional transgenic lines that define specific regions of the inner ear, including non‐sensory epithelial structures, would be advantageous, facilitating the analysis of regional cell behaviours.

One way of identifying new transgenic marker lines is through random insertion, where lines of interest can be selected on the basis of expression pattern. Identification of the insertion site of the transgene can reveal both the genomic enhancer sequences controlling transgene expression and the endogenous gene or genes that they regulate. Many PCR‐based techniques are available for mapping insertion sites; examples include inverse PCR (Brown et al., 2022), linker‐mediated PCR (Ellingsen et al., 2005; Wu et al., 2003), or amplification between transgene‐specific primers and degenerate primers (Liu & Whittier, 1995; Ma & Zhang, 2015; Parinov et al., 2004). Sequence‐based approaches can also be used, but require a high coverage to be certain to find the region of interest. This problem can be overcome by combining cross‐linking, proximity ligation, and selective amplification to target the insertion region prior to DNA sequencing (De Vree et al., 2014; Hottentot et al., 2017).

We describe two enhancer trap lines generated by random insertion that form useful markers for characterising epithelial morphogenesis in the developing zebrafish inner ear. We have used both PCR‐based and Targeted Locus Amplification approaches to identify the insertion sites for these transgenes. We show them to be driven by regulatory sequences for *smad6b*, which codes for a BMP regulator, and *fzd1*, which codes for a Wnt receptor. These lines provide useful tools for imaging the developing zebrafish ear and understanding the morphogenetic events that happen during the formation of the semicircular canal system.

## 2 MATERIALS AND METHODS

### 2.1 Animals

Zebrafish (*Danio rerio*) were housed at 28.5°C in the University of Sheffield or King's College London aquarium facilities, following standard husbandry methods and guidelines (Aleström et al., 2020; Westerfield, 2007). The wild‐type lines used were AB (ZDB‐GENO‐960809‐7) and King's Wild Type (KWT). Enhancer trap lines used were *Et*(*fzd1:EGFP*) and *Et(smad6b: EGFP‐CAAX)* (this work), together with the transgenic line *Tg(Xla.Eef1a1:h2b‐mRFP1)* (referred to throughout as *Tg(xEF1A: H2B‐RFP)*) (Rodríguez‐Aznar et al., 2013). Embryos were raised at 28.5°C in E3 medium (5 mM NaCl, 0.17 mM KCl, 0.33 mM CaCl_2_, 0.33 mM MgSO_4_, pH 7.2) containing 0.0001% methylene blue. Embryos were staged according to Kimmel et al. (Kimmel et al., 1995).

### 2.2 Generation of the *Et(smad6b:EGFP‐CAAX)* line

The *Et(smad6b: EGFP‐CAAX)* line was generated in an enhancer trap screen by ligating a 4.1 kb fragment upstream of the zebrafish *en2a* coding region to a minimal *c‐fos* (*fosab*; zfin.org) promoter driving membrane‐(CAAX)‐tagged EGFP in the Gateway vector pDestTol2, generating the construct en2acfospromEGFPMycpDest. This was injected into the KWT strain using the Tol2 system (Kawakami, 2007). Injected embryos were grown to adulthood and their offspring were selected on the basis of GFP expression in the ear.

### 2.3 Generation of the *Et(fzd1:EGFP)* line

The *Et(fzd1:EGFP)* line was originally generated during the establishment of a transgenic zebrafish assay for enhancer activity, using the T2K‐gata2‐EGFP‐C1 destination vector (Ishibashi et al., 2013), generating the construct T2K‐gata2‐EGFP‐miR‐137. Fish were selected that showed expression of GFP (cytoplasmic) in the ventral pillar of the developing otic vesicle.

### 2.4 Thermal asymmetric interlaced (TAIL)–PCR

Thermal Asymmetric Interlaced (TAIL)–PCR (Liu & Whittier, 1995; Parinov et al., 2004) alternates a higher annealing temperature for Tol2‐specific primers with a lower annealing temperature for degenerate primers designed to bind to the genomic sequence. This approach was used to generate short fragments flanking the transgene insertion site. TAIL–PCR products from secondary and tertiary nested PCR reactions were separated by gel electrophoresis, and fragments with the correct size shift—corresponding to the distance between the nested Tol2 primer sites—were isolated and sequenced. For the *Et(smad6b: EGFP‐CAAX)* transgene, Tol2‐specific and degenerate (AD) primer sequences used were: Tol2 5′‐1, GGGAAAATAGAATGAAGTGATCTCC; Tol2 5′‐2 GACTGTAAATAAAATTGTAAGGAG; Tol2 5′‐3 CCCCAAAAATAATACTTAAGTACAG; Tol2 3′‐1 CTCAAGTACAATTTTAATGGAGTAC; Tol2 3′‐2 ACTCAAGTAAGATTCTAGCCAGA; Tol2 3′‐3 CCTAAGTACTTGTACTTTCACTTG; AD‐3 WGTGNAGNANCANAGA; AD‐5 WCAGNTGWTNGTNCTG; AD‐6 STTGNTASTNCTNTGC; AD‐11 NCASGAWAGNCSWCAA, as described (Liu & Whittier, 1995). Sequences from either side of the insertion site were mapped to the zebrafish GRCz11 genome assembly. For the *Et(smad6b: EGFP‐CAAX)* line, two fragments (Tol2 5′‐3/AD11 and Tol2 3′‐3/AD5) were identified from the 5′ and 3′ ends of the insertion site at Ch18:19,702,439 (GRCz11), at the 3′ end of the *smad6b* locus (+3378 bp). A third fragment was also identified that mapped to a second site on chromosome 16.

### 2.5 Targeted locus amplification (TLA)

Targeted Locus Amplification (TLA) (Cergentis B.V., Utrecht, Netherlands (De Vree et al., 2014; Hottentot et al., 2017)) is a cross‐linking‐based technique that enables the selective amplification of local genomic sequence surrounding a transgene insertion. For cell preparation from zebrafish embryos, the protocol was adapted as follows. For each transgene, two hundred zebrafish embryos at the 15–20 somite stage were dissociated in a clean Petri dish under a coverslip in a minimal volume of Ca^2+^‐free Ringer's solution, as previously described (Baxendale et al., 2009). Cells were collected in a 1.5 mL reaction tube and separated from yolk platelets by centrifugation at 300 g for 1 min. The cell pellet was gently resuspended in Accumax™ cell dissociation medium (Invitrogen/Thermofisher) and incubated until cells were fully dissociated. DNA from ~10^7^ cells was crosslinked using the Cergentis kit, before shipping to Cergentis B.V. (www.cergentis.com) for TLA analysis.

Two insertion sites were identified for *Et(smad6b: EGFP‐CAAX)*, which matched the sites identified by TAIL–PCR, and one site was identified for *Et(fzd1:EGFP)*. Insertion sites were amplified by PCR between a genome‐specific primer and a Tol2‐specific primer on both sides of each insertion, using the following genome‐specific primers: *smad6b* locus: F: GGGTTAGGGGTAGGAAAGGAATA, R: GCAAACATACCCACGTTGCTAT; *crvpn1l* locus: F: GGACATATCACCTAAATCCGCTG, R: GTACAATAAATACACCTCAATG; *fzd1* locus: F: TTGCGCAAACATGTTGAAAAGTG, R: CTCTTTAGCAGCACCCAATGTAAA. PCR fragments were sequenced to confirm the insertion sites.

### 2.6 In situ hybridisation

Embryos were fixed by incubating in 4% paraformaldehyde overnight at 4°C. Antisense probe synthesis and in situ hybridisation were carried out as described (Thisse & Thisse, 2008). In some samples, embryo pigment was removed after the fixation step by incubation in a bleaching solution (PBS, 3% H_2_O_2_, 0.5% KOH) for 30 min. Probes were synthesised for the following genes: *crvpn1l, fzd1, gfp, smad6b, cdk14*; see Table S1.

### 2.7 Microscopy and image analysis

Images of in situ hybridisation stains were taken on an Olympus BX51 compound microscope equipped with differential interference contrast (DIC) optics, a C3030ZOOM camera, and CellB software, or a Micropublisher 6 camera and Ocular software.

For fluorescence imaging of live samples, larvae were anaesthetised with 4% tricaine and mounted in 1% low‐melting point agarose in E3 medium in a glass‐bottomed 35 mm Petri dish. Still images were taken on a Zeiss LSM880 Airyscan microscope in FAST‐SR Airyscan mode using 10×/0.3 N.A. (air), 20×/0.75 N.A. (air) or 40×/1.2 N.A. (water immersion) objectives or a Nikon CSU‐W1 spinning disk confocal microscope using 10×/0.45 N.A. and 20×/0.75 N.A. objectives. Airyscan images were processed using Zen Black 2.3 SP1 software using Auto Airyscan processing settings.

Time‐lapse imaging was performed using a Zeiss Z.1 light‐sheet microscope and ZEN Black 2014SP1 software using a 20×/1.0 N.A. water immersion objective with 1.0× or 1.5× optical zoom. Embryos were mounted vertically (either head up or head down) in 0.8% low melting point agarose in E3 medium in glass capillaries and imaged at 28.5°C in E3 medium with 4% tricaine as an anaesthetic in the imaging chamber. Images were acquired every 5 min using 5% 488 nm and 5% 561 nm laser power, 99.9 ms exposure time, light sheet thickness of 4.49 μm, and a *z*‐interval of 1 μm.

For multiview imaging in the Zeiss Z.1 light‐sheet microscope, embryos were mounted in 0.8% agarose containing a 1/8000 dilution of red fluorescent beads (0.5 μm, Sigma). *Z*‐stacks encompassing the whole depth of the sample were acquired from four different angles (at 90° intervals) using a 20×/1.0 N.A. water immersion objective and 0.36× optical zoom, 2.5% 488 nm and 2.0% 561 nm laser power, 99.9 ms exposure time, light‐sheet thickness of 7.32 μm and a *z*‐interval of 1.652 μm. Multiview images were registered using landmark alignment in Zen Black 2014SP1 software. The registered datasets were then fused and deconvolved using mean fusion followed by fast‐iterative deconvolution using PSFs extracted from the bead channel. Fused, deconvolved datasets were 3D rendered using arivis Vision4D (v3.4.0).

Images were processed in Fiji (Schindelin et al., 2012) and fluorescence images were assembled using the QuickFigures Plugin (Mazo, 2021). Figures were compiled using Adobe Photoshop v22.5.1 or Adobe Illustrator. All the images are lateral views with anterior to the left unless otherwise stated.

## 3 RESULTS AND DISCUSSION

### 3.1 A *smad6b*:EGFP Enhancer trap line marks cells of the zebrafish otic epithelium

**FIGURE 1:**
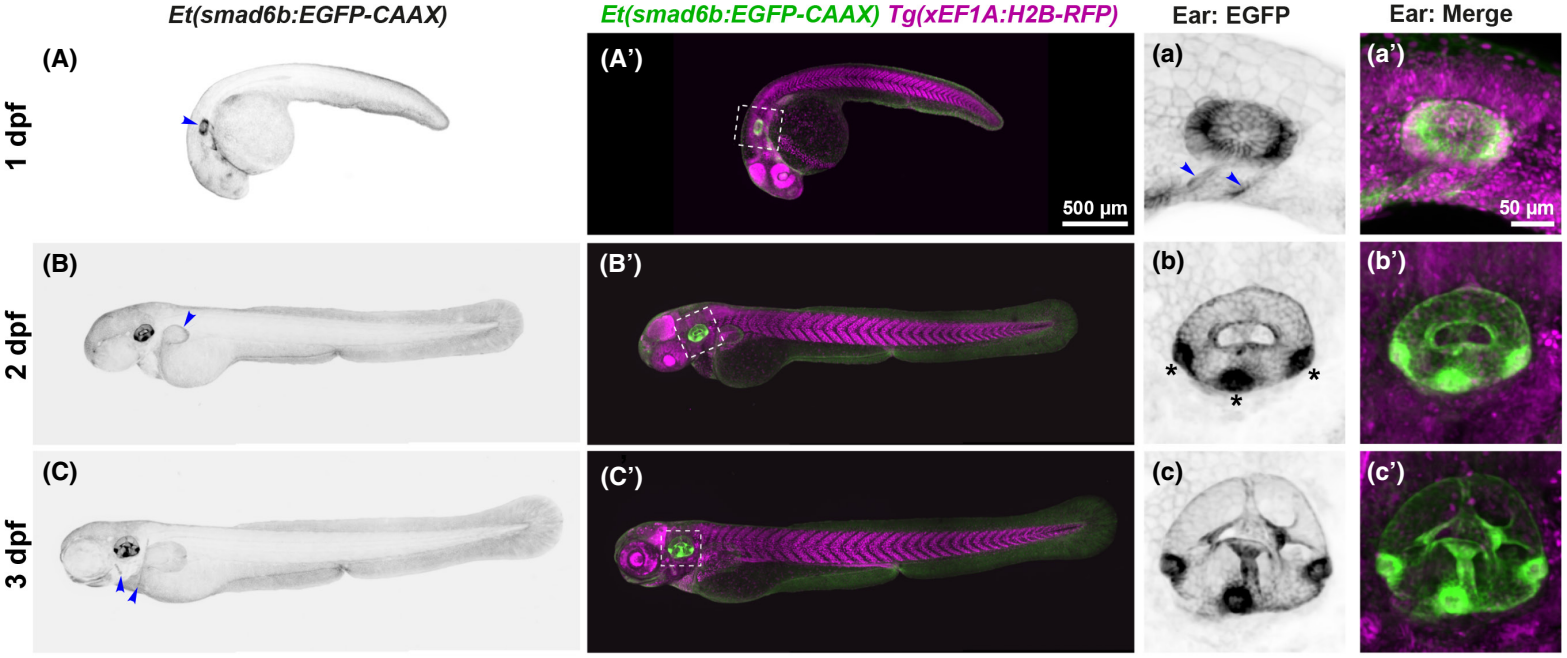
Expression of the *Et(smad6b: EGFP‐CAAX)* line. (A–C′) Whole mount images of *Et(smad6b:EGFP‐CAAX)*; *Tg*(*xEF1A:H2B‐RFP*) double transgenic embryos taken with a Nikon CSU‐W1 spinning disk confocal microscope at 1, 2 and 3 days post fertilisation (dpf); lateral views, anterior to the left. Arrowhead in (A) marks the otic vesicle. Panels (a–c') show maximum intensity projection (MaxIP) enlargements of EGFP expression in the otic vesicle at 1–3 dpf, corresponding to the white boxes in (A'–C′). Strong expression is present in the three cristae (b, asterisks). EGFP expression is also seen in the pharyngeal region at 24 hpf (a, arrowhead) and in the developing pectoral fin (B, arrowhead) at all stages. Lower level expression is present throughout the skin at all stages and in the forming opercle and cleithrum at 3 dpf (C, arrowheads). The *Tg*(*xEF1A: H2B‐RFP*) line provides a counterstain for all nuclei (magenta). Scale bars: 500 μm in (A'), for (A–C′); 50 μm in (a') for (a–c').

**FIGURE 2:**
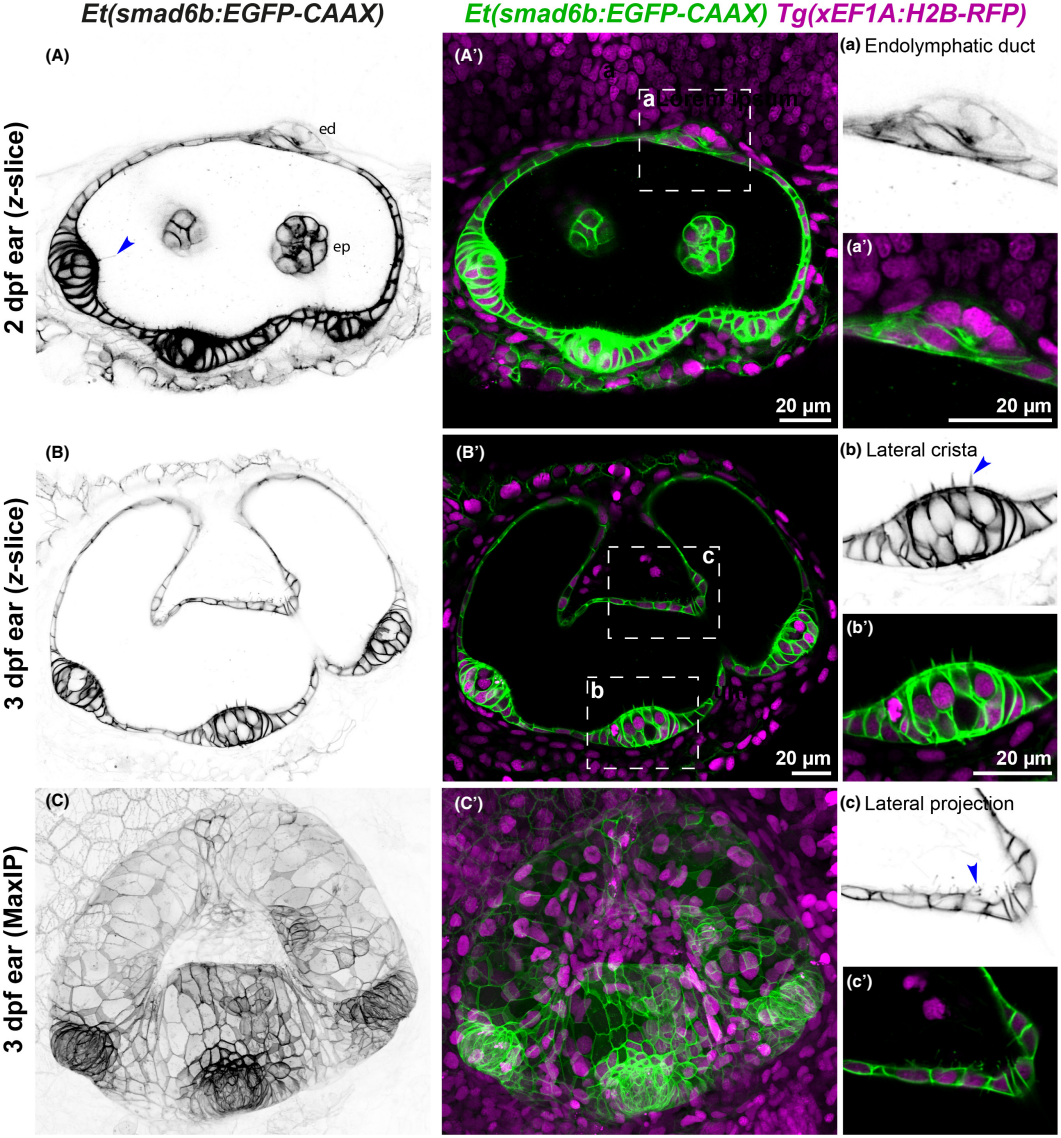
*Et(smad6:EGFP)* expression reveals cell shape details in the inner ear. (A–C′) Images of the otic vesicle of *Et(smad6b:EGFP‐CAAX)* embryos taken with a Zeiss Airyscan microscope at 2 and 3 dpf; lateral views, anterior to the left. (A–B′) Single *z*‐slice images at 2 dpf (A, A'), 3 dpf (B, B′) and Maximum Intensity Projection (MaxIP) of 3 dpf (C, C′). Panels (A–C) show inverted images of EGFP expression; (A–C′) show *Et(smad6b: EGFP‐CAAX)* expression in green and *Tg*(*xEF1A: H2B‐RFP*) expression in all nuclei in magenta. The inverted image reveals details such as the kinocilia (A, arrowhead) on crista hair cells. Regions of interest highlighting cellular details (white boxes on the middle images) are enlarged on the right with the inverted green channel (a–c) and merged channels (a'–c'). (a, a') Endolymphatic duct emerging from dorsal otic epithelium. (b, b') Lateral crista showing pseudostratified epithelium and hair cells with stereocilia (b, arrowhead). (c, c') Part of the lateral projection showing membranous protrusions from the basal surface of otic epithelial cells (c, arrowhead). Abbreviations in (A): ed, endolymphatic duct; ep, epithelial projection. Scale bars: 20 μm in A', for A; 20 μm in B′, for B, C, C′; 20 μm in a', for a; 20 μm in b' for b, c, c'.

The *Et(smad6b: EGFP‐CAAX)* enhancer trap line expresses GFP in most cells of the otic epithelium (Figures 1 and 2; Movie S1). Expression in the otic vesicle is present from 24 h post fertilisation (hpf) and remains throughout development, more strongly on the lateral side of the ear. As the otic vesicle grows, the GFP expression level varies; fluorescence in the three cristae is particularly bright and much stronger than in the two maculae (Figure 1b–c'). Single *z*‐slices reveal that cells in the tightly packed pseudostratified epithelia of the cristae have stronger expression compared with surrounding non‐sensory epithelium and with the thin squamous cells of the dorsal otic epithelium (Figure 2b–b'). GFP expression is also present in cells of the endolymphatic duct (Figure 2a–a') and the epithelial projections and pillars that undergo morphogenesis to define the semicircular canal ducts (Figure 1c,c'; Figure 2a). Occasional otic epithelial cells lack expression of GFP, particularly in the anterior macula. In addition to the strong otic expression, weaker expression is seen in the pharyngeal region and fins, with general low‐level expression in the skin (Figure 1; Movie S1). This line provides an excellent tool for imaging otic epithelia in the live zebrafish embryo at all stages of embryogenesis and can be used for the 3D analysis of cell shape (Mendonca et al., 2021).

The *Et(smad6b: EGFP‐CAAX)* enhancer trap line was generated by random insertion of a construct consisting of a fragment of zebrafish *en2a* upstream sequence and a minimal *c‐fos* promoter driving expression of membrane‐tagged EGFP (EGFP‐CAAX) (Figure S1). Some transgenic fish showed GFP expression in the ear and were isolated on this basis. To identify the insertion site of the transgene, we used a Thermal Asymmetric Interlaced (TAIL)–PCR‐based approach (Liu & Whittier, 1995) (see Materials and Methods). This identified an insertion site in the 3′ region of the *smad6b* locus on Chromosome 18 and a second potential site on Chromosome 16 (Figure S1). To confirm these results, we also used Targeted Locus Amplification (TLA; Cergentis) (De Vree et al., 2014; Hottentot et al., 2017) (see Materials and Methods). Two integration sites were found: one at Ch18:19,702,439 (GRCz11), confirming the *smad6b* insertion site, and the second site at Ch16:49,012,534 (GRCz11) within the *cysteine‐rich venom protein natrin‐1‐like* (*crvpn1l*) gene. Both insertion sites were confirmed by PCR (Figure S2).

**FIGURE 3:**
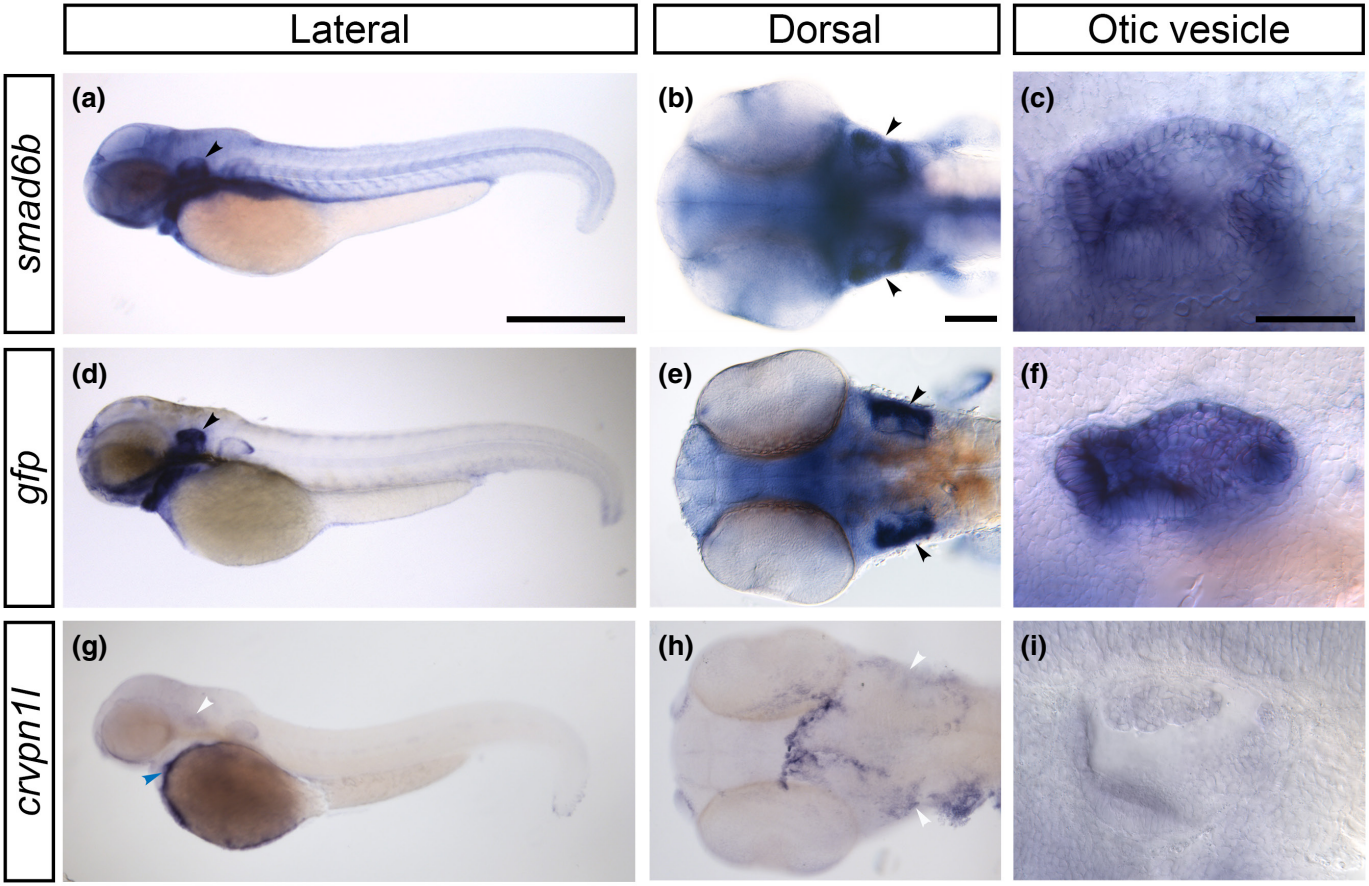
Comparison of *smad6b* and *crvpn1l* expression in wild‐type embryos w the expression of the *gfp* transgene in *Et(smad6b: EGFP‐CAAX)* transgenic embryos. (a–i) Whole mount in situ hybridisation at 48 h post fertilisation (hpf) showing *smad6b* expression in wild‐type embryos (a–c), *gfp* expression in *Et(smad6b:EGFP‐CAAX)* embryos (d–f), and *crvpn1l* expression in wild‐type embryos (g–i); anterior to the left. (a, d, g) Lateral views of the whole embryo. Note the similar expression domains for *smad6b* (a) and *gfp* (d), with relatively strong *gfp* expression in the ear (marked by black arrowheads). Expression of *crvpn1l* (g) is absent in the ear (white arrowheads), but is present over the yolk sac (blue arrowhead). (b, e, h) Dorsal view of the head at 48 hpf showing expression of *smad6b* (b) *gfp* (e) and *crvpn1l* (h). Note the closely matching spatial expression domains of *smad6b* and *gfp*, with stronger expression laterally in the otic vesicles (d, e, arrowheads). (h) Dorsal view of the head showing expression of *crvpn1l* at 48 hpf, in a different pattern to that of the *gfp* mRNA (compare with [e]). (c, f, i) Detailed lateral views of the otic vesicle, showing the matching expression of *smad6b* (c) and *gfp* (f) throughout the otic vesicle. No otic expression is seen for *crvpn1l* (I). Scale bars: 500 μm in (a), for (d, g); 100 μm in (b), for (e, h); 50 μm in (c), for (f, i).

To provide further validation of the insertion sites, we used in situ hybridisation to examine the endogenous expression patterns of the transcripts from each candidate gene and compared these with the expression of the transgene. Both the GFP fluorescence and *gfp* mRNA expression patterns recapitulated the endogenous expression of *smad6b* mRNA, including expression in the otic vesicle, most strongly on the lateral side (Figure 3). Endogenous *smad6b* expression appeared to be unaffected in homozygous transgenic embryos. We did not detect any GFP expression that recapitulates the *crvpn1l* expression pattern, as assayed by in situ hybridisation (Figure 2), and did not find any offspring with a GFP expression pattern that differed from the *smad6b* expression; the reason why this second insertion site has been retained is unknown. We conclude that the GFP fluorescence derives from transgene expression at the *smad6b* locus, and have named the line *Et(smad6b: EGFP‐CAAX)*.

The Smad6 protein is an intracellular inhibitor of BMP signalling (Hata et al., 1998; Imamura et al., 1997). Zebrafish have two *smad6* genes: both *smad6a* (Mowbray et al., 2001) and *smad6b* (Thisse & Thisse, 2005) are expressed in the lateral epithelium of the zebrafish otic vesicle at 24 hpf. The precise function of *Smad6* during otic morphogenesis has yet to be addressed. In the chick otocyst, electroporation of *Smad6* has been used to demonstrate a requirement for canonical BMP/pSMAD signalling in cell thinning to form the dorsolateral pouch that gives rise to the anterior and posterior semicircular canal ducts (Ohta et al., 2010), the dorsolateral expression of *Dlx5* (Ohta et al., 2016), and the acquisition of non‐sensory (septum cruciatum) fates in the crista (Chang et al., 2008). Given its normal expression in the ventrolateral otic epithelium in the chick (Ohta et al., 2010), Smad6 may function to restrict canonical BMP/pSMAD signalling to dorsolateral regions of the otocyst in this species. Smad6b is also thought to mediate the regulation of BMP signalling by Notch during zebrafish angiogenesis (Mouillesseaux et al., 2016).

### 3.2 A *frizzled1*:EGFP Enhancer trap line marks the ventral epithelial projection and pillar of the developing zebrafish ear

**FIGURE 4:**
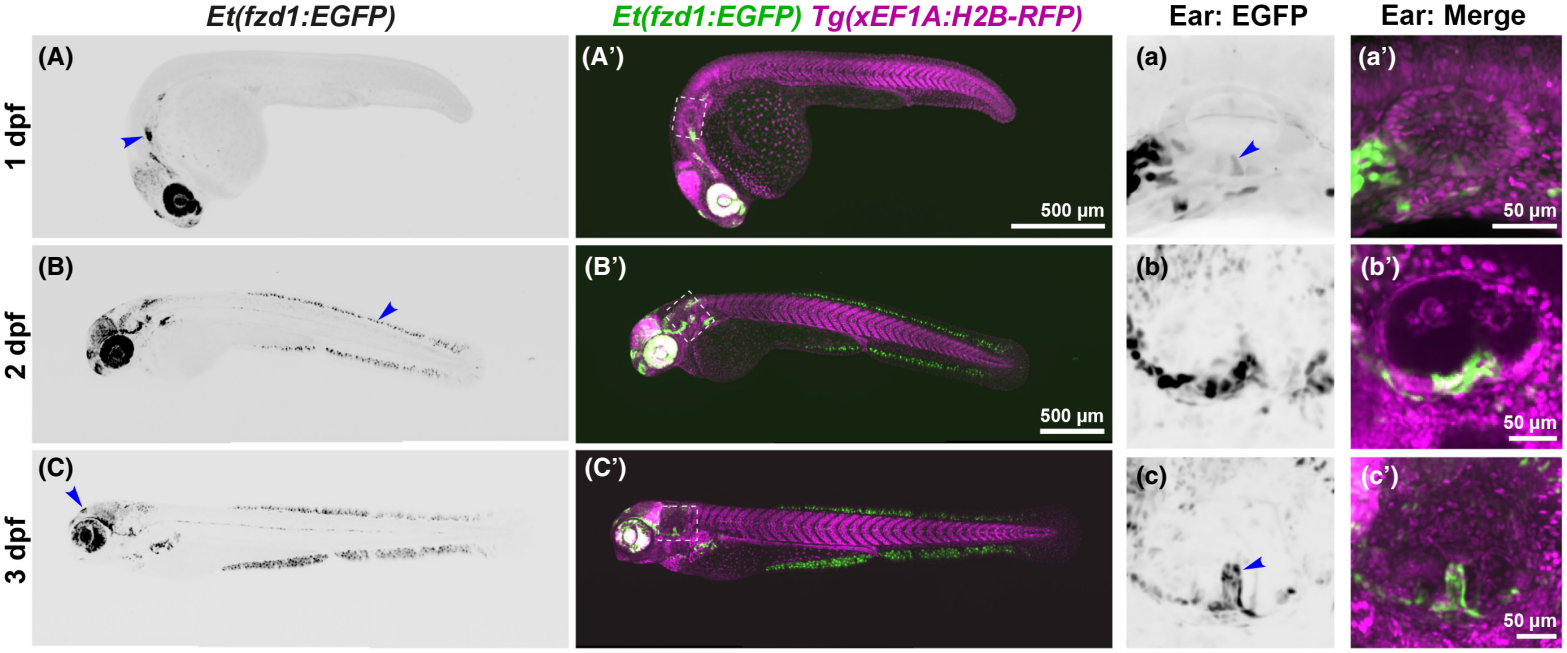
Expression of the *Et(fzd1:EGFP)* line. (A–C') Whole mount images of *Et(fzd1:EGFP)* embryos taken with a Nikon CSU‐W1 spinning disk confocal microscope at 1, 2, and 3 dpf, anterior left. (a–c') Enlarged images of the otic vesicle at 1–3 dpf, corresponding to the white boxes in (A'–C′). Panels (A–C) and (a–c) are MaxIP inverted images of the EGFP expression; (A'–C′) and (a'–c') show the merged channels, with *Et(fzd1:EGFP)* expression in green and *Tg*(*xEF1A:H2B‐RFP*) expression in all nuclei in magenta. All are MaxIP images with the exception of (b'), which is a single *z*‐slice to show the GFP expression in ventral otic epithelium. (A–a') At 1 dpf, GFP expression is strongest in the retina and lens of the eye, and anterior (A, arrowhead) and posterior lateral line ganglia, with weaker expression in the midbrain and forebrain. Occasionally, some embryos also have expression in the epiphysis and heart. Expression in the otic vesicle at 1 dpf is limited to one or two cells (a arrowhead). (B–b') At 2 dpf, strong staining persists in the eye, and midbrain expression increases. New expression in the dorsal and ventral fins is strong in a subset of cells (B, arrowhead). In the otic vesicle, *Et(fzd1:EGFP)* expression is strongest in a ventral domain (b, b'). (C–c') At 3 dpf, expression of *Et(fzd1:EGFP)* is maintained in the fins and reduced in the eye and brain. Expression in the epiphysis can still be seen in some embryos (C, arrowhead). In the otic vesicle, most cells of the ventral pillar are marked by GFP expression (c, arrowhead; see also Figure 5). Scale bars: 500 μm in (A'), for (A); 500 μm in (B′), for (B′, C, C′); 50 μm in (a') for (a), in (b) for (b') and in (c) for (c').

**FIGURE 5:**
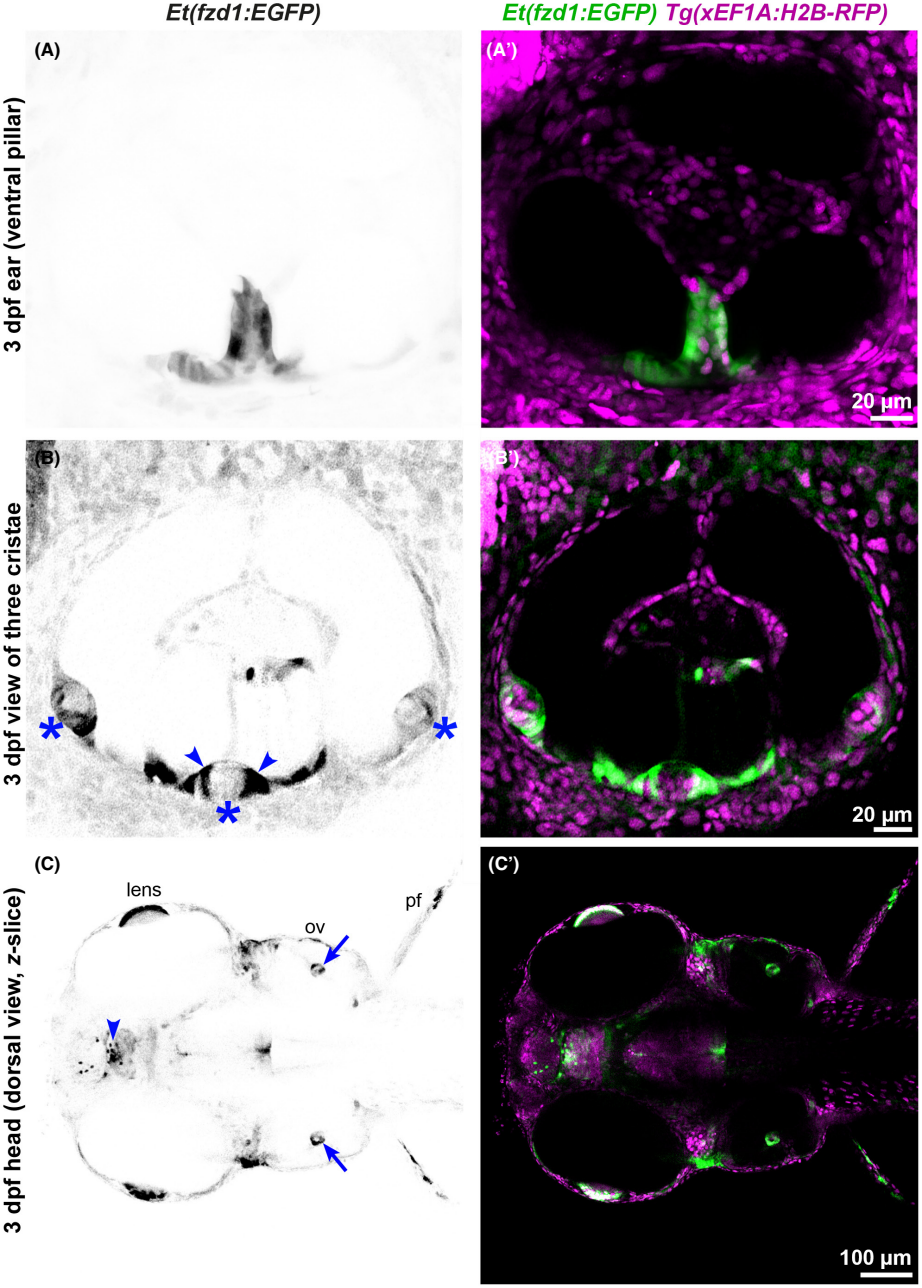
Expression of the *Et(fzd1:EGFP)* line in the developing inner ear. (A–C′) Images of the otic vesicle of *Et(fzd1:EGFP)* embryos taken with a Nikon CSU‐W1 spinning disk confocal microscope at 3 dpf; lateral views with anterior to the left. All are single *z*‐slice images taken at different focal planes. (A–C) Inverted images of GFP expression. (A'–C′) Merged channel images showing *Et(fzd1:EGFP)* expression in green and *Tg*(*xEF1A:H2B‐RFP*) expression in all nuclei in magenta. (A, A') Lateral view of the ventral pillar, marked by expression of GFP. (B, B′) GFP expression in the three cristae (B, asterisks) is strongest in the supporting cells at the margins of the lateral crista (B, arrowheads; compare to the *fzd1* mRNA expression in Figure 6f, inset). (C, C′) Dorsal view of the head at the level of the ventral pillar (C, arrows mark expression in the ventral pillars; compare to the *fzd1* mRNA expression in Figure 6e). GFP is also expressed in the lens of the eye, epiphysis (C, arrowhead) and pectoral fins. Abbreviations in C: ov, otic vesicle; pf, pectoral fin. Scale bars: 20 μm in (A'), for (A); 20 μm in (B′), for (B); 100 μm in (C′) for (C).

We have also characterised a second enhancer trap line, fortuitously generated during establishment of a zebrafish reporter assay for human enhancer activity (Ishibashi et al., 2013). Occasionally, random insertion of the transgenic constructs can show position or enhancer trap effects (Ishibashi et al., 2013). In this case, transgenic fish were selected on the basis of GFP‐positive cells in the ventral pillar of the ear at 72 hpf (Figures 4 and 5; Movie S2), reflecting a likely enhancer trap event. GFP expression was variable between individuals, even those that were homozygous for the transgene. In addition to expression in the ear, GFP fluorescence was variably present in cells in the brain, the epiphysis, in a cluster of cells just dorsal to the trigeminal ganglion, in the heart, lens, and retina of the eye, and fins (Figure 4). In the otic vesicle of transgenic embryos, GFP was expressed in cells within the ventral otic epithelium from 24 hpf, and later in the ventral projection and pillar (Figure 5). Expression was also present at the margins—but not in the central (hair cell) zone—of the lateral crista, with a similar but weaker expression pattern in the anterior and posterior cristae (Figure 5).

We initially attempted to use TAIL–PCR to identify the insertion site for this transgene, but this was unsuccessful, most likely because the insertion site turned out to be in a repetitive site close to a telomere (see Figure S3). We next turned to the TLA approach. Sequencing reads revealed that the transgene is located on chromosome 16 at position 53,924,905 (GRCz11), ~5.8 kb upstream of *frizzled 1 (fzd1)* and ~ 94.5 kb downstream of *cyclin‐dependent kinase 14 (cdk14)* (1.2 kb downstream of a predicted *cdk14* variant exon) (Figure S3). To confirm the position of the insertion site, we amplified fragments from either side of the insertion site using genomic DNA from transgenic embryos and a Tol2‐specific primer, and a genomic sequence‐specific primer.

**FIGURE 6:**
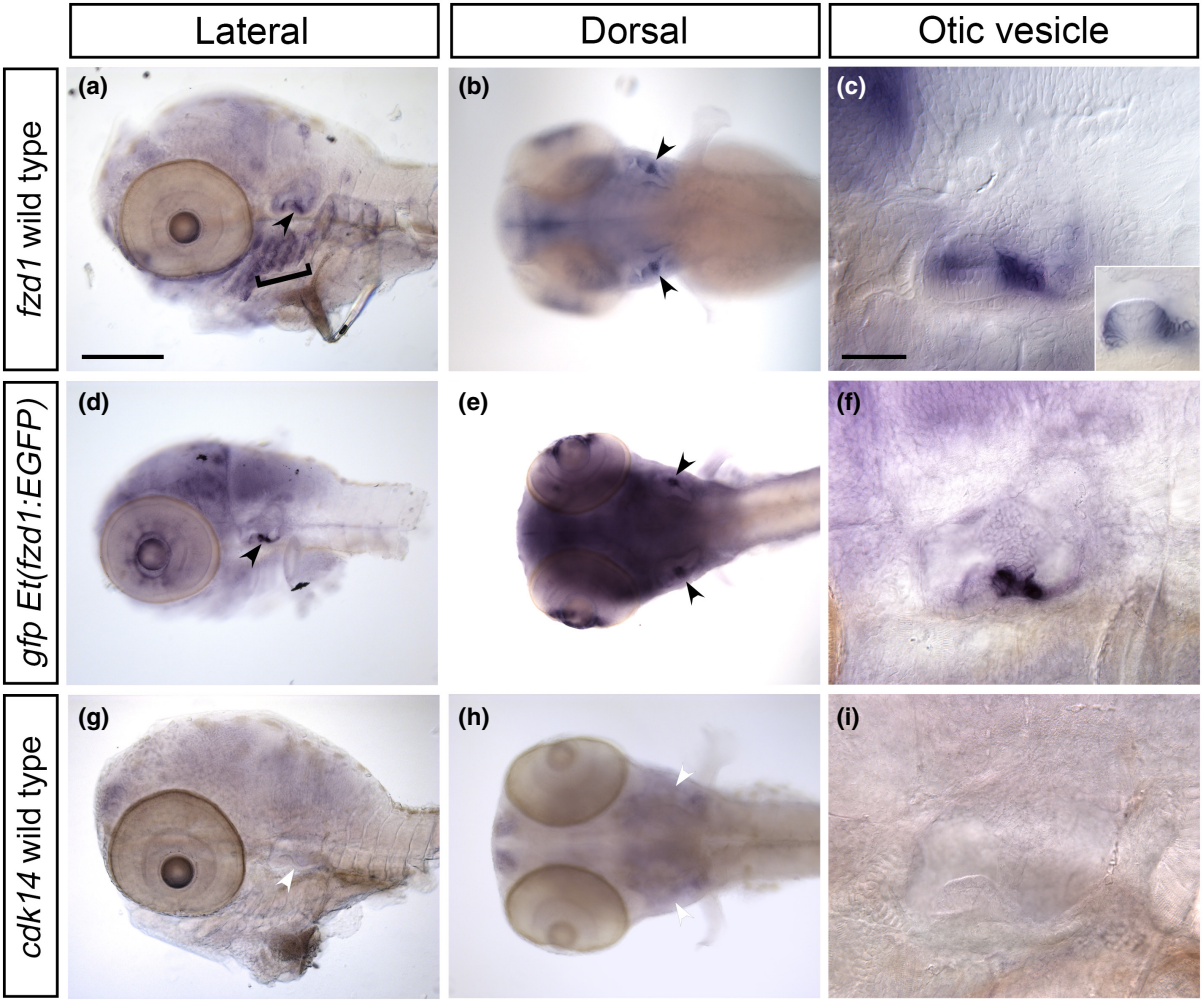
Comparison of *fzd1* and *cdk14* mRNA expression in wild‐type embryos to expression of *gfp* mRNA in *Et(fzd1:EGFP)* transgenic embryos. (A–I) Whole mount in situ hybridisation at 3 dpf, showing *fzd1* expression in wild‐type embryos (A–C), *gfp* expression in *Et(fzd1:EGFP)* embryos (D–F) and *cdk14* expression in wild‐type embryos (G–I); anterior to the left. (A, D, G) Lateral views of the head. Note the closely matching spatial expression domains of *gfp* and *fzd1* genes in the otic vesicle (black arrowheads), whereas *cdk14* is not expressed in the otic vesicle (white arrowheads). Expression of *fzd1* is also present in the pharyngeal region in wild‐type embryos (A, bracket), but *gfp* expression is absent in this region in the *Et(fzd1:EGFP)* line (D). (B, E, H) Dorsal view of the head, clearly showing the ventral pillar expression (black arrowheads) in the ear of both *fzd1* (B) and *gfp* (E). Expression of both *fzd1* and *gfp* is also seen in part of the retina. (C, F, I) Detailed lateral views of the otic vesicle, showing the matching expression of *fzd1* (C) and *gfp* (F) in the ventral pillar. Inset in C at a different focal plane shows expression of *fzd1* at the lateral margins of the lateral (horizontal) crista, matching the GFP fluorescence shown in Figure 5b,b'. No expression in either the ventral pillar (H, white arrowheads) or lateral crista (I) is seen for *cdk14*. Scale bars: 200 μm in (A), for (B, D, E, G, H); 50 μm in (C), for (F, I).

To validate the insertion site further, we generated antisense RNA probes to *fzd1*, *gfp*, and *cdk14*, and used these to compare expression of these genes by in situ hybridisation, and to the GFP fluorescence. Expression of *fzd1* mRNA in wild‐type embryos is a close temporal and spatial match to both the *gfp* mRNA and GFP expression pattern in transgenic embryos in the brain and ear (Figure 6). GFP expression in the epiphysis and the fins, however, is not matched by *fzd1* expression (Figure 6); these domains might reflect a leaky expression of the transgene or expression driven by the human enhancer sequences in the construct. It is also possible that detection of the GFP fluorescence is more sensitive than the detection of transcripts by the chromogenic in situ hybridisation reaction. In addition, *fzd1* expression in the pharyngeal region is not recapitulated by the transgene expression (Figure 6). Expression in the ear, however, is a close match, with both *fzd1* mRNA and GFP expressed in cells at the margins of the lateral crista and at the base of the ventral pillar (Figure 6). GFP fluorescence in the ventral epithelial projection and pillar appears to extend further than the *fzd1* expression detected in fixed embryos by in situ hybridisation; this may be due to collapse of the pillar in fixed samples or might result from persistence of the green fluorescent protein in this tissue in living embryos. In contrast to *fzd1*, expression of *cdk14* does not match that of the *gfp* mRNA or GFP fluorescence (Figure 6). We conclude that the transgene is located on chromosome 16 and that the otic expression is driven by *fzd1* regulatory sequences, and have named the line *Et(fzd1:EGFP)*.

In zebrafish, *fzd1* expression has only previously been characterised at earlier embryonic stages (Nikaido et al., 2013). Frizzled proteins are G protein‐coupled receptors for the Wnt signalling pathway; in mammals, Wnt signalling is known to have multiple roles in the development of the inner ear, including dorsal patterning of the otocyst (Noda et al., 2012; Riccomagno et al., 2005), resorption of cells at the semicircular canal fusion plate (Rakowiecki & Epstein, 2013), and in the planar cell polarity of sensory hair cells in the cochlea (Yu et al., 2010). Expression of *Frizzled1* mRNA has been reported in the otocyst of chick (Sienknecht & Fekete, 2009) and mouse (Borello et al., 1999; Durruthy‐Durruthy et al., 2014), and at later stages in the mammalian cochlea (Daudet et al., 2002; Yu et al., 2010). It is not yet known which Wnt acts as a ligand for Fzd1 in the zebrafish ear, although Wnt3, facilitated by the co‐receptor Lrp5, interacts with Fzd1 in the zebrafish brain (Veerapathiran et al., 2020). Transcripts for *wnt3* are expressed in the zebrafish ear, at least from 4 days post fertilisation (4 dpf) (Clements et al., 2009); *wnt4* is also expressed in ventral otic epithelium (Thisse & Thisse, 2005).

## 4 CONCLUSION

Enhancer trapping provides an unbiased approach to generating reporter lines for the live imaging of specific cell types. This technique can have different advantages compared with the more recent targeted approaches that use gene editing techniques to knock‐in reporter genes at a specific locus (see, for example, Wierson et al., 2020). Random insertion of a transgene can lead to variable domains or levels of expression compared with the endogenous gene, which may be advantageous or provide useful new markers. In fact, both the lines described here show enhanced expression of GFP in the otic vesicle relative to other tissues, compared with the endogenous gene expression. Although the disadvantage of this approach is the need to map insertion sites, this can be a worthwhile endeavour to uncover new genes expressed in a tissue of interest.

**FIGURE 7:**
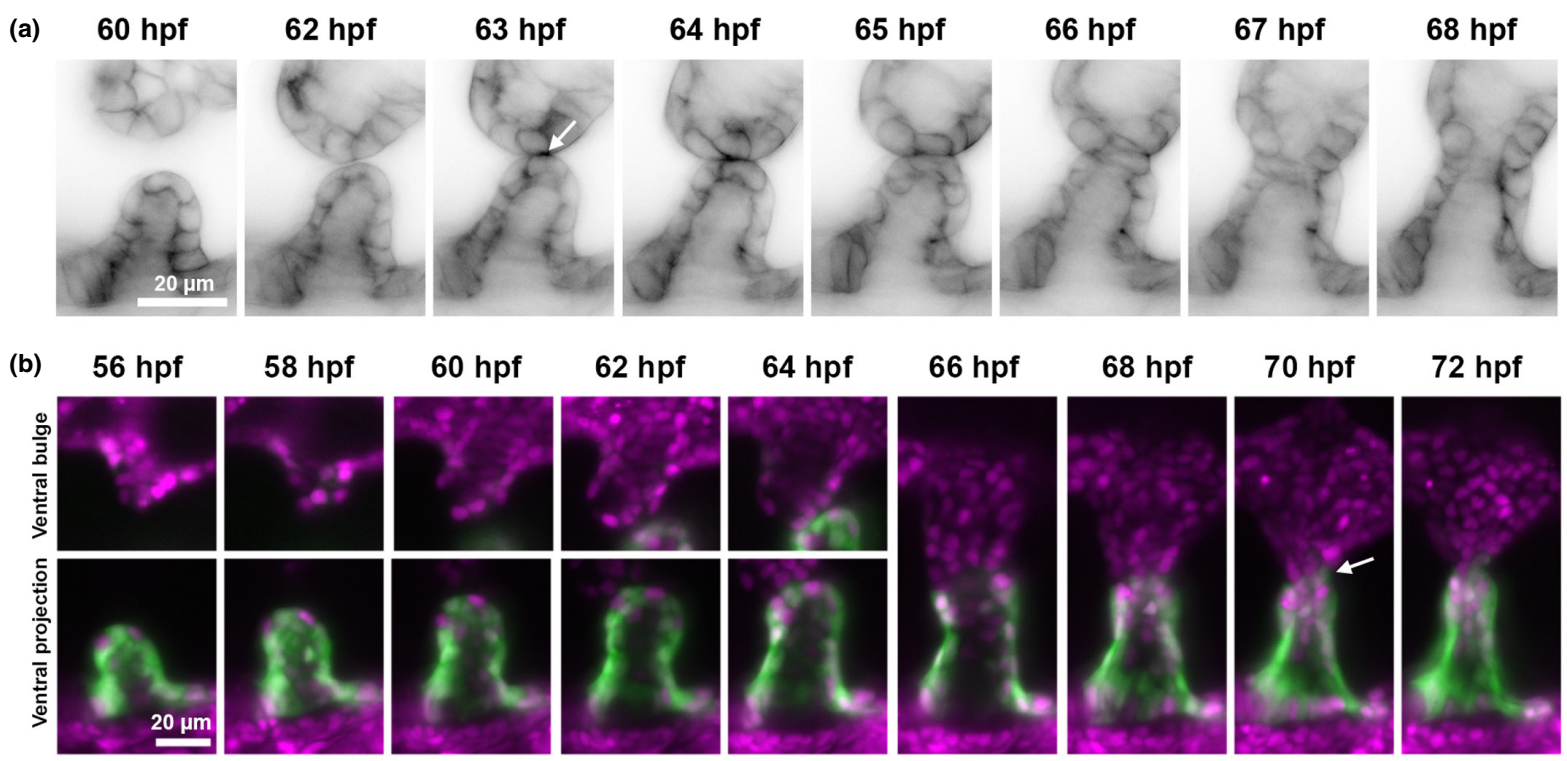
Imaging of ventral pillar formation in *Et(smad6b:EGFP‐CAAX)* and *Et(fzd1:EGFP)* ears. (A, B) Series of stills from time‐lapse movies of *Et(smad6b:EGFP‐CAAX)* (A, inverted greyscale) and *Et(fzd1:EGFP)* (B, green), showing the meeting and fusion of the ventral projection and the ventral bulge of the lateral projection to form the ventral pillar in the otic vesicle. *Et(smad6b:EGFP‐CAAX)* images clearly show the initial contact of cell membranes (A, arrow, 63 hpf). The *Et(fzd1:EGFP)* line marks the ventral projection but not the ventral bulge: GFP‐labelled and unlabelled cells can be seen meeting at the fusion plate (B, arrow, 70 hpf). *Tg(xEF1A: H2B‐RFP)* expression (magenta) marks all nuclei. Scale bars: 20 μm in (A) (applies to all panels); 20 μm in (B) (applies to all panels).

The *Et(smad6b:EGFP‐CAAX)* and *Et(fzd1:EGFP)* enhancer trap lines will both be useful for following different aspects of ventral pillar morphogenesis in the developing zebrafish inner ear. Light‐sheet imaging of the *Et(smad6b:EGFP‐CAAX)* line reveals the cell–cell contact between cells of the ventral projection and cells of the lateral projection ventral bulge at 63–64 hpf (Figure 7). This line will allow characterisation of the dynamic changes in cell shape and behaviour at the fusion plate or in other regions of the projection and bulge. This is complemented by the *Et(fzd1:EGFP)* line, where cytoplasmic GFP expression distinguishes cells originating in the ventral epithelial projection from cells originating in the ventral bulge of the lateral projection (Figure 7), and can be used to follow cell trajectories before, during and after the fusion of these structures. Initial descriptions of the epithelial fusion and perforation events of pillar formation in the zebrafish were based on electron microscopy (Waterman & Bell, 1984) or conventional histology (Haddon & Lewis, 1996); the use of live imaging will now make it possible to determine the origin and fate of individual cells within the pillar.

In summary, the *Et(smad6b:EGFP‐CAAX)* and *Et(fzd1:EGFP)* lines are useful tools for live imaging of the otic vesicle in the developing zebrafish embryo. Our results also demonstrate that Targeted Locus Amplification is a robust method for the rapid identification of the genomic insertion sites of randomly‐inserted transgenes, even where the insertion site is close to a telomere or in repetitive sequence, and should be widely applicable.

## AUTHOR CONTRIBUTIONS

DB and SB performed the experimental work and data analysis, with imaging support from NvH and technical support from MM and EG. The *Et(smad6b: EGFP‐CAAX)* line was originally generated and provided by RK; the *Et(fzd1:EGFP)* line was originally generated by FS‐G, in the lab of T.S. Becker (University of Sydney). DB, SB, NvH and TTW prepared the figures. DB, SB and TTW designed the study and wrote the article.

## FUNDING INFORMATION

This work was funded by the Biotechnology and Biological Sciences Research Council (BBSRC: BB/J003050 to TTW, BB/M01021X/1 and BB/S007008/1 to TTW and SB, and BB/D020433/1 to RDK). DB was funded by an Anatomical Society Ph.D. studentship. Light‐sheet imaging was carried out in the Sheffield Wolfson Light Microscopy Facility, supported by a BBSRC ALERT14 award (BB/M012522/1) to TTW and SB. The funders had no role in study design, data collection and analysis, decision to publish, or preparation of the article.

## CONFLICT OF INTEREST STATEMENT

The authors have no conflicts of interest to declare.

## LICENCING

For the purpose of open access, the authors will apply a Creative Commons Attribution (CC BY) licence to any Author Accepted Manuscript version arising from this study.

## Supporting information


Figure S1.

Figure S2.

Figure S3.

Movie S1.

Movie S2.

Table S1.

Baldera et al Supplementary Figure and Movie legends.

## Data Availability

The data that support the findings of this study will be made available on reasonable request. The *Et(smad6b:EGFP‐CAAX)* and *Et(fzd1:EGFP)* lines will be maintained as live fish and/or preserved as cryogenic sperm samples at the University of Sheffield and will be made available on reasonable request.
