# Supplementary figures and images for: Enhancer trap lines with GFP driven by smad6b and frizzled1 regulatory sequences for the study of epithelial morphogenesis in the developing zebrafish inner ear
Source: J Anat. 2023 Feb 6;243(1):78–89. doi: 10.1111/joa.13845 (PMC10273346; doi:10.1111/joa.13845)

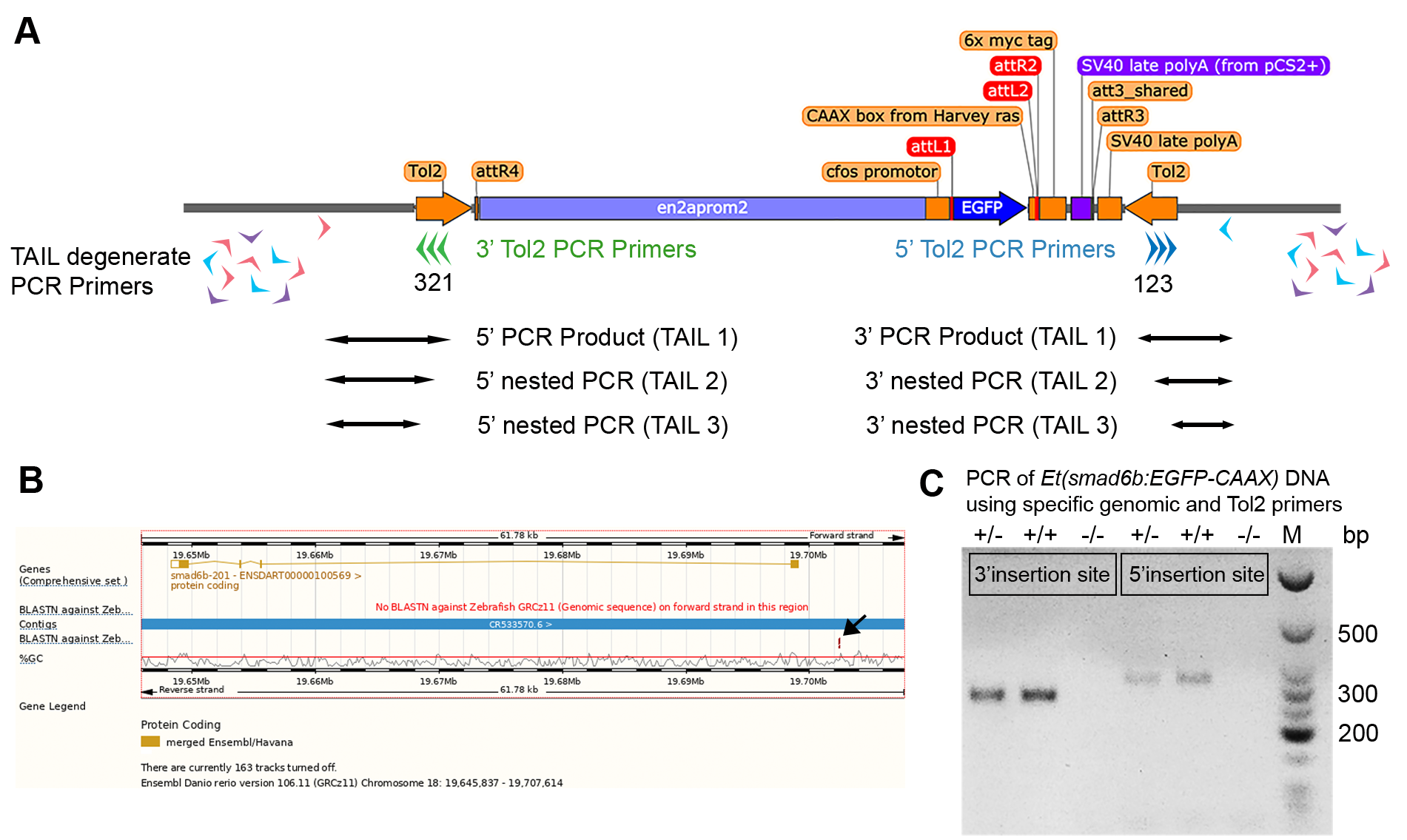

Supplement: Supplementary file 1 — Figure S1. [file JOA-243-78-s004.tif]

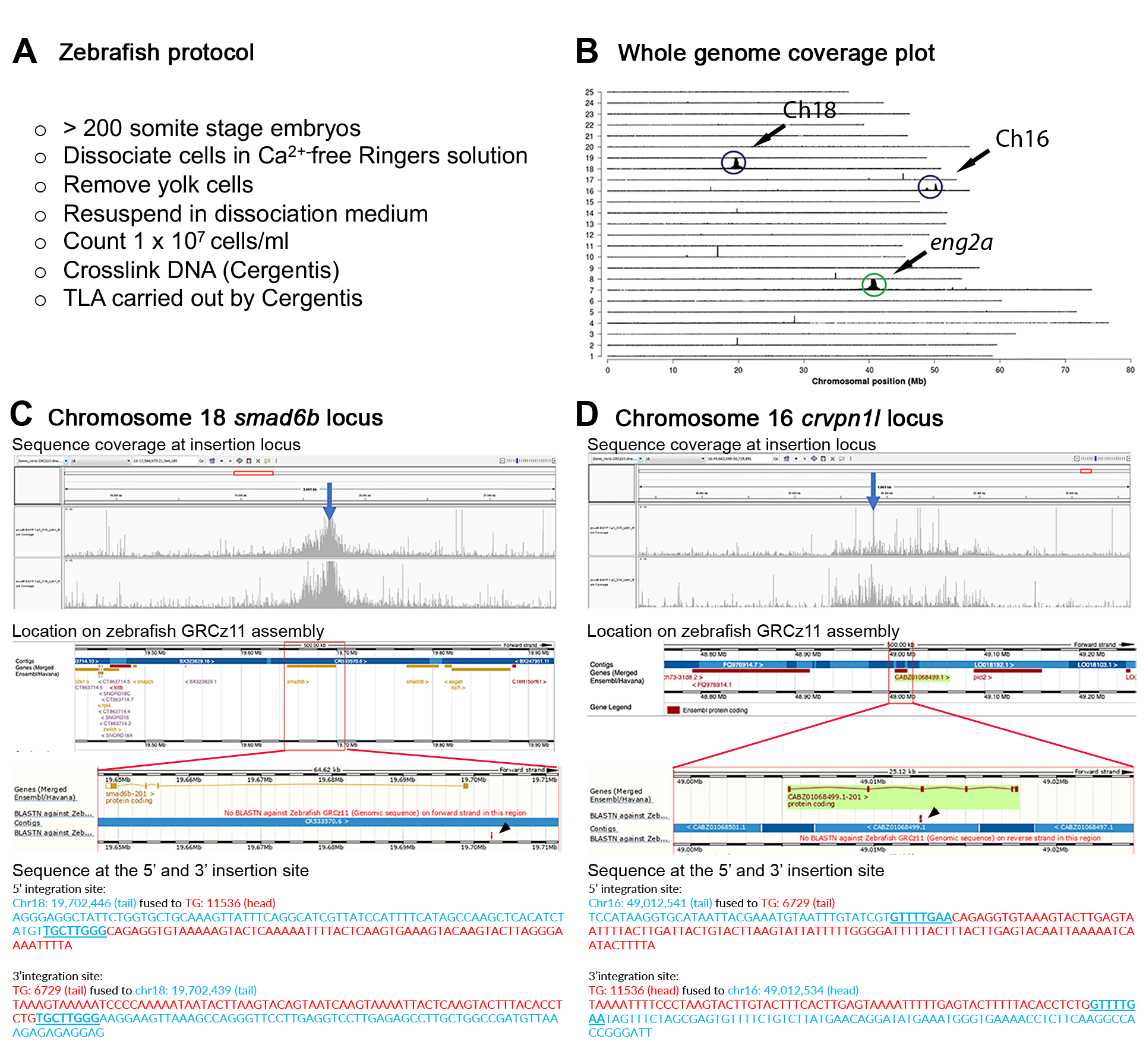

Supplement: Supplementary file 2 — Figure S2. [file JOA-243-78-s001.tif]

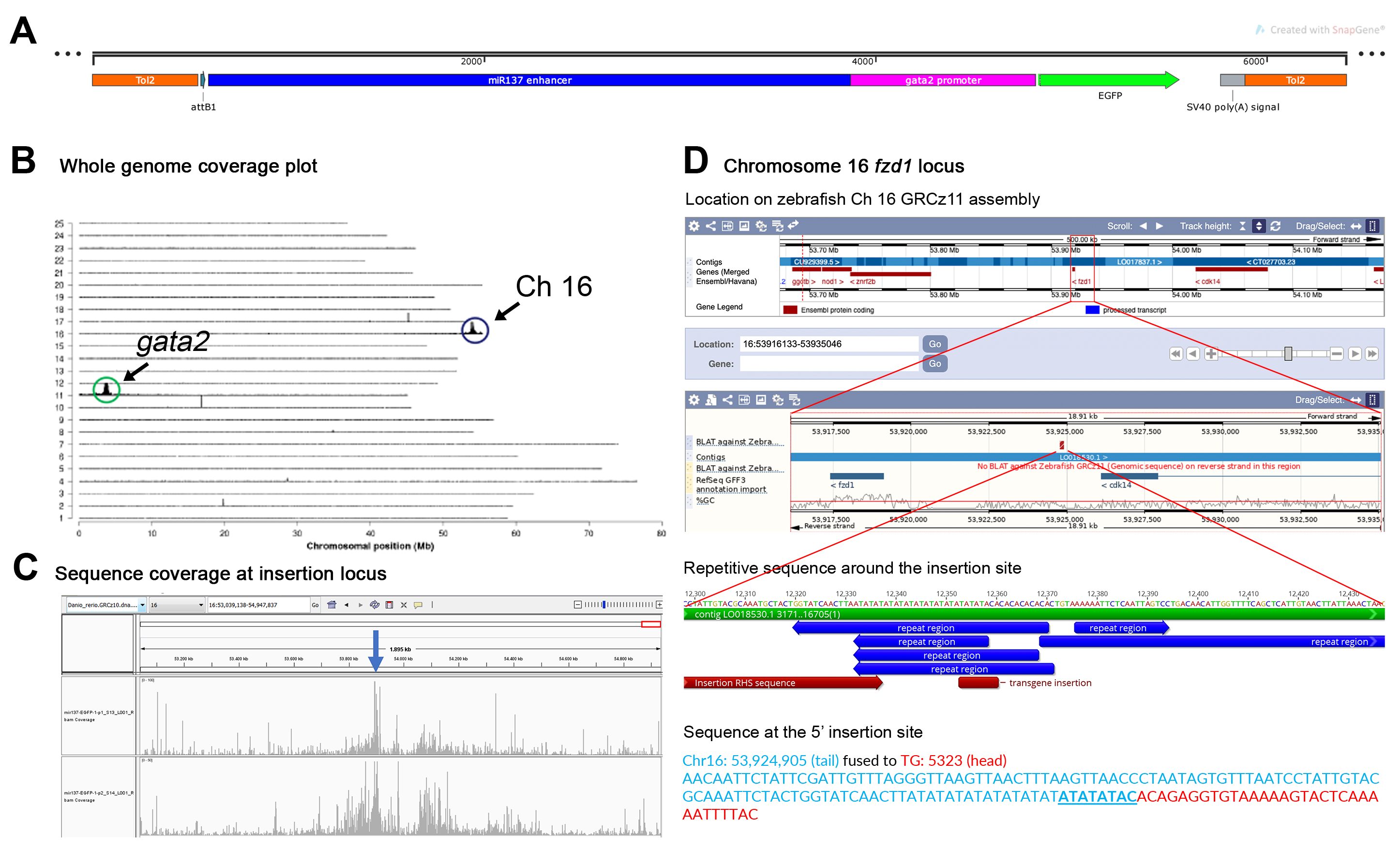

Supplement: Supplementary file 3 — Figure S3. [file JOA-243-78-s006.tif]
