# Supplementary material for: Enhancer trap lines with GFP driven by smad6b and frizzled1 regulatory sequences for the study of epithelial morphogenesis in the developing zebrafish inner ear
Source: J Anat. 2023 Feb 6;243(1):78–89. doi: 10.1111/joa.13845 (PMC10273346; doi:10.1111/joa.13845)
Supplement: Supplementary file 6 — Table S1. [file JOA-243-78-s003.docx]

**Supplementary Table 1 List of probes for *in situ* hybridisation**

| **Gene** | **ZFIN ID (zfin.org)** | **Primer sequences (all shown 5’>3’)** |
| --- | --- | --- |
| *cdk14* | ZDB-GENE-080220-44 | F, GAACGCCGACATGTGTGATTTA  R, ACGTCATTTTCTTGTCTTGATAGCT |
| *crvpn1l* | CABZ01068499.1 | F, GACTCATATGAACAGCTGGACACAAG  R, GCCTTTTCCATGACATGATGAAAGTA |
| *fzd1* | ZDB-GENE-990415-219 | F, AGACTCTTTCTCACCGCACACCG  R, AGCAGTAATAATAACGAAAGCTGAGC |
| *smad6b* | ZDB-GENE-050419-198 | F, ATGTTCAGGACGAAACGCTCAGGTC  R, GCTCCTTGCCTGAGCATGGACG |
