# Supplementary material for: Enhancer trap lines with GFP driven by smad6b and frizzled1 regulatory sequences for the study of epithelial morphogenesis in the developing zebrafish inner ear
Source: J Anat. 2023 Feb 6;243(1):78–89. doi: 10.1111/joa.13845 (PMC10273346; doi:10.1111/joa.13845)
Supplement: Supplementary file 7 — Baldera et al Supplementary Figure and Movie legends. [file JOA-243-78-s007.docx]

**Enhancer trap lines with GFP driven by *smad6b* and *frizzled1* regulatory sequences for the study of epithelial morphogenesis in the developing zebrafish inner ear**

Davide Baldera^1§*^, Sarah Baxendale^1§^, Nicholas J. van Hateren^1^, Mar Marzo^1^, Emily Glendenning^1^, Fan-Suo Geng^2**^, Kazutomo Yokoya^3^, Robert D. Knight^3^, Tanya T. Whitfield^1#^

^1^School of Biosciences, University of Sheffield, Sheffield, S10 2TN, UK

^2^Brain and Mind Research Institute, University of Sydney, Sydney, New South Wales, Australia

^3^Centre for Craniofacial and Regenerative Biology, King’s College London, Guy’s Hospital, London SE1 9RT, UK

^§^These authors contributed equally and share first authorship

**Current affiliations:**

*CeSASt, University of Cagliari, Monserrato, Cagliari, Italy

**Data Science Institute, The University of Technology Sydney, Sydney, Australia

^#^Corresponding author:

Tanya T. Whitfield: [t.whitfield@sheffield.ac.uk](mailto:t.whitfield@sheffield.ac.uk)

**Supplementary Figure and Movie Legends**

**SUPPLEMENTARY FIGURE S1 Identification of the genomic insertion site of *Et(smad6b:EGFP-CAAX)* using TAIL-PCR.**

**A** Overview of the Thermal Asymmetric Interlaced (TAIL-PCR) strategy. Schematic structure of the transgene, containing *engrailed2a* promoter sequences, a *c-fos* minimal promoter and EGFP-CAAX coding sequence, flanked by Tol2 sequences. Tol2-specific primers are shown for the 5’ (green) and 3’ (blue) Tol2 sites. Degenerate primers are shown in red, blue and purple. PCR products from reactions using specific Tol2 primers and degenerate primers are shown. The products from nested PCR reactions (TAIL2 and TAIL3) were used to find flanking genomic sequences.  **B** PCR products from 5’ and 3’ reactions that map to the same position in the genome were found to match sequences at the 3’ end of the *smad6b* locus. Black arrow shows the position of sequences around the insertion site. **C** The *smad6b* insertion site was confirmed using specific genomic primers and Tol2 primers with DNA from embryos containing one copy (+/-), two copies (+/+) or no copies (-/-) of the transgene. Amplicons of 270 bp (3’ to insertion site) and 310 bp (5’ to insertion site) were confirmed by DNA sequencing. M, low molecular weight DNA ladder (NEB).

**SUPPLEMENTARY FIGURE S2 Identification of the genomic insertion site of *Et(smad6b:EGFP-CAAX)* using Targeted Locus Amplification.**

**A** Protocol for preparation of zebrafish cell suspension for the Targeted Locus Amplification (TLA) method. **B** Result of TLA sequence trace mapping to the zebrafish genome. Peaks can be seen at the same Ch18 locus close to *smad6b*, and on Ch16, as found by TAIL-PCR. The peak on Ch7 in the green circle corresponds to the endogenous *eng2a* locus. **C** Detailed view of the sequence traces around the *smad6b* insertion site above an overview of the genomic location. Note *smad6b* is also close to *smad3b*. Below are the insertion sequences at the 3’ and 5’ insertion sites. Note the target site duplication (TSD) 8 bp tandem repeated sequence, underlined in bold. **D** Detailed view, as in **C**, for the Ch16 insertion site. The insertion is in the intronic sequence of the *cysteine-rich venom protein natrin-1-like* (*crvpn1l*) gene. Both insertion site sequences are mapped to the zebrafish GRCz11 assembly, and the position numbers shown correspond to the positions in GRCz11; however, the original genome coverage plot and sequence coverage data is to the zebrafish GRCz10 assembly.

**SUPPLEMENTARY FIGURE S3 Identification of the insertion site for the *Et(fzd1:EGFP)* line using Targeted Locus Amplification.**

**A** Schematic structure of the construct used to generate the *Et(fzd1:EGFP)* transgene, containing zebrafish *gata2* promoter sequences, a human *miR-137* enhancer (Giacomotto et al., 2016) and the EGFP coding sequence, flanked by Tol2 sequences. **B** Result of TLA sequence trace mapping to the zebrafish genome. On the whole genome coverage plot, peaks can be seen near a telomere of Ch16, close to *fzd1*. The peak on Ch11 in the green circle corresponds to the endogenous *gata2* locus. **C** Sequence coverage around the insertion site shows a strong peak (blue arrow) with some gaps to the right of the peak due to repetitive sequences. **D** Overview of the genomic location on Chromosome 16; the insertion site is upstream of *fzd1* and downstream of a *cdk14* variant exon. The lower panel shows the DNA sequence around the insertion site and the many repetitive sequences; the insertion site is within a simple AT repeat. Note the target site duplication (TSD) 8 bp tandem repeated sequence, underlined in bold. Insertion site sequences are mapped to the zebrafish GRCz11 assembly, and position numbers shown correspond to the positions in GRCz11; the original genome coverage plot and sequence coverage data were to the zebrafish GRCz10 assembly.

**SUPPLEMENTARY MOVIE 1** **3D rendering of *Et(smad6b:EGFP-CAAX)* expression in 4 dpf embryo.**

3D rendering of multiview light-sheet imaging of *Et(smad6b:EGFP-CAAX)* expression in a 4 dpf zebrafish larva. *Z*-stacks taken at 90° rotation intervals were fused and deconvolved to generate a single *z*-stack. *Et(smad6b:EGFP-CAAX)* expression is shown in white; high expression levels are seen in the otic epithelia, with lower levels of expression in the skin. 3D rendering and animation was performed with arivis Vision4D.

**SUPPLEMENTARY MOVIE 2** **3D rendering of *Et*(*fzd1:EGFP*) expression in 3 dpf embryo.**

3D rendering of fused and deconvolved multiview light-sheet imaging of *Et(fzd1:EGFP)* expression in a 3 dpf zebrafish larva. *Et(fzd1:EGFP)* expression (green) is seen in the ventral pillar, the eye and the midbrain. *Tg(xEF1A:H2B-RFP)* expression (magenta) marks all nuclei.

**REFERENCE**

**Giacomotto, J., Carroll, A. P., Rinkwitz, S., Mowry, B., Cairns, M. J. and Becker, T. S.** (2016). Developmental suppression of schizophrenia-associated miR-137 alters sensorimotor function in Zebrafish. *Transl. Psychiatry* **6**, e818.
